# Intra-cellular lactate concentration in T lymphocytes from septic shock patients — a pilot study

**DOI:** 10.1186/s40635-018-0167-4

**Published:** 2018-02-05

**Authors:** Thibaut Girardot, Thomas Rimmelé, Guillaume Monneret, Julien Textoris, Fabienne Venet

**Affiliations:** 1 Hospices Civils de Lyon, Anesthesia and Critical Care Medicine Department, Edouard Herriot Hospital, Lyon, France; 2 Hospices Civils de Lyon, Immunology Laboratory, Edouard Herriot Hospital, Lyon, France; 3 EA 7426 (Université Claude Bernard Lyon 1) “Pathophysiology of Injury-Induced Immunosuppression–PI3”, Edouard Herriot Hospital, Lyon, France; 4 Joint Research Unit (bioMérieux–Hospices Civils de Lyon–Université Claude Bernard Lyon 1), Edouard Herriot Hospital, Lyon, France; 5 Laboratoire Commun de Recherche Hospices Civils de Lyon–bioMérieux, Hôpital Edouard Herriot, Pavillon P, 5ème étage, 5, place d’Arsonval, 69003 Lyon, France

**Keywords:** Sepsis, Lymphocyte, Lactate, Warburg effect, Bioenergetics, Technique optimization

## Abstract

**Background:**

Sepsis-associated hyperlactatemia is a widely used biomarker, associated with initial severity and poor outcomes. This increased circulating lactate concentration has been proposed to result in part from a mismatch between oxygen delivery and demand in organs. However, other mechanisms may participate. In particular, a metabolic reprogramming similar to the Warburg effect initially described in cancer cells could lead to increased lactate production by immune cells such as T lymphocytes after sepsis. The objective of this study was to set up a protocol for lactate measurement in T lymphocytes, and to evaluate whether lactate production by T lymphocytes was increased in septic shock patients.

**Methods:**

We first optimized protocols for lactate and pyruvate measurements in T lymphocytes purified from healthy volunteers’ blood, either stimulated with phytohaemagglutinine (PHA) or left untreated. We then conducted a pilot study to confirm the feasibility of this protocol in samples from septic shock patients.

**Results:**

PHA stimulation induced aerobic glycolysis in human lymphocytes ex vivo, with increased lactate and pyruvate productions. To correctly measure this phenomenon, minimal cell number was 250,000 and optimal culture duration was 40 h. We also observed a significant correlation between lactate concentration in T lymphocytes and in their culture supernatants. We were able to measure lactate concentration in T lymphocytes from septic shock patients. Our preliminary results showed that intra-lymphocyte lactate concentration was not different between patients and healthy volunteers.

**Conclusion:**

This protocol should now be tested in a larger cohort of patients. The association between immune cell metabolic reprogramming as measured by lactate concentration in T cells and functionality represents an exciting field for research.

## Background

Sepsis definition has recently been updated as a life-threatening organ dysfunction caused by a dysregulated host response to an infectious process. Its most severe form, septic shock, is characterized by profound circulatory, cellular, and metabolic abnormalities [1]. Septic shock is diagnosed by the association of a sepsis-related hypotension in the absence of hypovolemia, leading to administration of vasopressors, and hyperlactatemia [1]. Incidence of severe sepsis syndromes is estimated at 270 per 100,000 person-years, and related hospital mortality at 26% (95% CI 20–33%), in high-income countries during the last decade. Thus, five million deaths per year worldwide are attributed to this syndrome [2]. However, the update of septic shock definition may affect these epidemiological data [3].

Injury-induced immune dysfunctions are supposed to be responsible for a large part of sepsis-related deaths [4, 5]. Recently, a link between an altered metabolism of innate immune cells and sepsis-induced immunoparalysis has been demonstrated [6], as well as the interplay between metabolism and trained immunity [7]. Such metabolic reprogramming in monocytes includes a switch towards aerobic glycolysis despite oxygen availability—the so-called Warburg effect—leading to increased intra-cellular lactate concentration [7].

During septic shock, the Th pattern of T lymphocytes shifts, which can be regarded as a metabolic adaptation as it is mediated in part by adrenergic stimulation [8]. In addition, immunometabolic alterations of circulating T cells have recently been described [9]. Lymphocytes could increase their lactate production through adrenergic stimulation, or through Warburg effect as observed in cancer cells [10–12]. Thus intra-cellular lactate concentration in T lymphocytes may reflect their metabolic adaptation to septic shock, whereas serum lactate level is a global marker of severity, tissue injury, and organ dysfunctions. Thus, these two parameters may not reflect similar dysfunctions during septic shock, although they may be linked to some extent. T lymphocytes bioenergetics reprogramming, as well as interactions between immunity and metabolism, are exciting fields for research and possible therapies [9].

Evidence of Warburg effect and increased lactate production in T lymphocytes during human sepsis is scarce. We postulated that lactate production by T lymphocytes may be increased after septic shock. We first determined optimal experimental conditions to measure lactate and pyruvate in healthy volunteers’ T cells with or without stimulation. We then confirmed its feasibility in a pilot study exploring lactate concentration in T cells purified from septic shock patients’ blood.

## Methods

### Blood samples and ethical concerns

Blood samples were provided by the French blood bank institution (“Etablissement Français du Sang”). All donors gave their explicit written consent for the use of their blood for biomedical research purposes, according to local legislation. Blood samples were collected in EDTA-anticoagulated tubes, anonymized upon donation, and were sent to the laboratory at room temperature, to be processed on the same day. Technical optimizations were performed on blood from eight healthy volunteers. Blood from 10 other healthy donors served as controls for comparison with septic shock patients’ blood. Median [Q1-Q3] age of these last 10 donors was 33 [30–40] years, and 50% were male.

Septic shock was defined according to the diagnostic criteria of the ACCPS/SCCM [13], as this study was conducted before the newest definitions were published [1]. Septic shock adult patients (*n* = 7) without pre-existing immunosuppressive disease or treatment were included in this study, as a part of a global study on injury-induced immune dysfunctions. It has been approved by our institutional ethical board (“Comité de Protection des Personnes Sud-Est II”, #IRB 11236). This study is also registered at the French Ministry of Research and Teaching (#DC-2008-509) and recorded at the national informatics committee (“Commission Nationale de l’Informatique et des Libertés”). Although the ethical board waived the need for informed consent—because the study was observational and measurements were made on residual blood samples after completion of routine follow-up—a non-opposition to inclusion in the study was recorded for each patient.

### T cell purification from whole blood

Purification of T lymphocytes from whole blood was performed through an antibody-based negative selection according to manufacturer’s instructions. A human T cell enrichment antibody cocktail (RosetteSep™, StemCell Technologies, Grenoble, France)—containing a mix of anti-CD16 (present on NK cells, monocytes, macrophages, and neutrophils), anti-CD19 (B lymphocytes), anti-CD36 (platelets, red blood cells, monocytes), anti-CD56 (NK cells), and anti-CD66b (granulocytes) antibodies—was added to the blood sample.

After incubation and centrifugation over density gradient medium (Biocoll®, Biochrom, Berlin, Germany), T lymphocytes were isolated. Residual red blood cells were lysed with Versalyse™ (Beckman Coulter, Brea, CA, USA). After purification, T cells were either processed directly to measure intracellular lactate concentration or cultured in complete culture medium, with or without stimulation (see below).

### T cell count and subset determination

At the end of the purification process, cells were counted with a flow cytometer (Navios, Beckman Coulter) using LDS (LDS 751, Molecular Probes, Life Technologies, Carlsbad, CA, USA) and calibration beads (Flow-Count™ Fluorospheres, Beckman Coulter).

Lymphocyte subset proportions were determined using a commercially available antibody mix containing anti-CD45, anti-CD3, anti-CD4, and anti-CD8 antibodies stained with fluorochromes (TetraChrome™ CD3-4-8-45, Beckman Coulter). Lymphocytes were identified on a size/CD45 plot then T cell purity was defined as the proportion of CD3+ cells among lymphocytes. T cell purity was routinely above 90%. Percentages of CD4+, CD8+, and CD4−/8− T cells were measured among CD3+ T cells.

Median [Q1–Q3] percentages were 65.8 [59.3–71.8] and 72.0 [59.9–80.9] % of CD4+, 28.2 [23.6–32.8] and 14.8 [11.0–26.5] % of CD8+, and 5.5 [4.4–7.1] and 3.1 [1.8–8.2] % of CD4−/8− T cells for healthy volunteers and septic shock patients, respectively. None of these differences were statistically significant; however, this could only be due to the low number of patients included in this study.

### Ex vivo culture and stimulation

In some experiments, purified T cells were diluted in RPMI (Roswell Park Memorial Institute) complete medium to achieve a concentration of 1 × 10^6^ cells/mL. RPMI complete medium is a RPMI 1640 medium (Eurobio, Les Ulis, France), supplemented with 10% human AB serum (Life Technologies), 200 μg/mL amphotericin B (Gibco), 1000 IU/mL penicillin (Eurobio), 1000 μg/mL streptomycin (Eurobio), and 200 mM L-glutamine (Eurobio).

Cells were then stimulated with 4 μg/mL PHA (phytohaemagglutinin, Remel-Oxoid, Dardilly, France) or left untreated (non-stimulated condition). Indeed, PHA stimulation is a standard mitogenic stimulus for T cell activation and induction of proliferation, and PHA-induced T cell functions are altered in septic shock patients [14, 15]. Duplicates were made whenever possible. Cell culture plates were then incubated in standard conditions (37 °C, 5% CO_2_). After a 40-h culture, supernatants were collected. Remaining cells were recovered after centrifugation and both samples were then deproteinised and stored at − 80 °C until metabolite measurement.

### Cell lysis and sample deproteinisation

Immediately after purification or cell culture, 250,000 T cells were aliquoted and lysed with the buffer supplied in the L-Lactate assay kit (ref ab65330, Abcam, Cambridge, UK). Efficacy of this cell lysis protocol was verified on two consecutive experiments using Trypan blue exclusion test (data not shown).

To avoid further modifications of lactate concentration in the samples due to the potential presence of enzymes, we decided to deproteinate the obtained cell lysates or cell culture supernatants. We used a chemical process, according to manufacturer’s instructions. The whole process took place on ice and in a cold 4 °C centrifuge. Perchloric acid was added to a final concentration of 1 M, resulting in protein denaturation. After centrifugation, supernatant was added with potassium hydroxide to normalize sample pH. This reaction produced potassium perchlorate, which was pelleted by centrifugation. The remaining deproteinised supernatant was collected and stored at − 80 °C. Efficacy of this deproteinisation protocol was checked with the Bradford technique (data not shown).

### Lactate and pyruvate quantitative determination

An enzymatic method followed by a colorimetric measure was used to determine lactate concentration in samples, with a commercially available kit (L-Lactate Assay Kit, ref. ab65330, Abcam).

T cell samples did not need any dilution, whereas supernatants were diluted 10 times before reading. A reaction mix prepared according to manufacturer’s instructions was mixed with the samples and incubated for 30 min. The emitted light was measured with a spectrophotometer (Victor™ X4 Multilabel Plate Reader, Perkin Elmer, Waltham, MA, USA) at a wavelength of 570 nm for 1 s. Samples and standard curves were always processed in duplicates. Means of duplicates were used to calculate lactate concentration in samples and standards. The mean measured luminescence of the blank, corresponding to the background luminescence, was subtracted to all measurements. Lactate concentrations were calculated from the standard curve (ranging from 0 to 10 nmol/well) generated from the lactate standard according to manufacturer’s instructions.

As lactate concentration was not detectable in samples containing 250,000 cells either from septic shock patients or healthy volunteers when measured by colorimetry, we used a fluorometric measurement (Ex/Em 540/590 nm) for this experiment. The Lactate Assay Kit (ref ab65330, Abcam) indeed allows for both measurement techniques, the fluorometric one being more sensitive for low lactate concentrations. This fluorometric measurement relies on the same basic principle than the colorimetric described above.

Pyruvate concentration was measured using a commercially available kit from the same manufacturer (Pyruvate Assay Kit, ref. ab65342, Abcam) followed by a fluorometric measurement (Ex/Em 540/590 nm).

### mHLA-DR, CD4+, and T regulatory lymphocytes count determination

These parameters were measured on whole blood by flow cytometry. HLA-DR expression on monocytes (mHLA-DR) was determined using the Quantibrite™ assay (ref 340827, BD Biosciences, Franklin Lakes, NJ, USA). HLA-DR-positive cells were selected among monocytes, identified as CD14+ cells. Results were expressed as numbers of antibodies bound per cells (ab/c) using standardized beads (BD Quantibrite™ beads) as described previously [16].

Absolute number of CD4+ lymphocytes and percentage of T regulatory cells were determined by a combined staining protocol with anti-CD4, anti-CD25, and anti-CD127 antibodies stained with fluorochromes. Calibration beads (Flow-Count™ Fluorospheres, Beckman Coulter) were added to be able to determine the absolute concentration of CD4+ lymphocytes, expressed as numbers of cells per μL of blood. Tregs were defined as CD4 + CD25highCD127−/low cells as described previously [17]. Percentage of Tregs was calculated among CD4+ cells.

### Statistical analyses

Means of the duplicates were used for all calculations. Statistical analyses were performed with R Studio® software (RStudio Inc., Boston, MA, USA). Comparisons between groups used non parametric Wilcoxon tests. Paired tests were performed for matched data. Differences with *p* values lower than 0.05 were considered statistically significant.

## Results

I. Technical optimization

1. Stimulation duration

We first determined the optimal stimulation duration to observe a difference in intracellular lactate concentration between non-stimulated and stimulated samples. For this first experience, we used 1 × 10^6^ T cells from four different healthy volunteers.

**Fig. 1:**
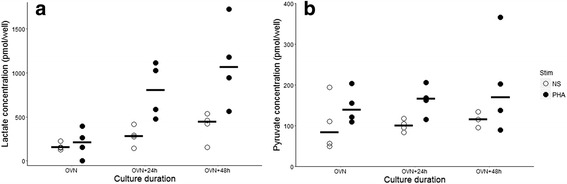
Optimal cell stimulation duration determination. T lymphocytes were purified from four healthy donors’ blood. Cells were cultured in complete RPMI medium at a concentration of one million cells/mL and were stimulated with 4 μg/mL PHA (black circles) or left unstimulated (NS, open circles). After various duration of incubation, ranging from overnight (OVN) to approximately 62 h (OVN + 48 h), one million T cells were aliquoted and intra-lymphocyte lactate and pyruvate concentrations were measured. Results are expressed as the quantity of lactate (Fig. 1**a**) or pyruvate (Fig. 1**b**) per well of the lecture plate, which contains 50 μL of the obtained cell lysate. Each point represents the mean of the duplicates for each donor, horizontal bars represent the median value

We observed that overnight stimulation (~ 16 h) did not result in any measurable increase in lactate concentration in purified T cells (Fig. 1a). A longer stimulation in culture led to a clear-cut increase in lactate concentration in PHA-stimulated T lymphocytes, compared to non-stimulated cells (Fig. 1a). This increase was measurable starting from 40 h (overnight + 24 h) of stimulation.

Regarding pyruvate, although time effect was less obvious, the same culture duration (overnight+ 24 h) also resulted in measurable difference between stimulated and non-stimulated samples (Fig. 1b). We thus decided to incubate T cells for 40 h (overnight + 24 h) before metabolite measurement.

2. Warburg effect in PHA-stimulated T lymphocytes

**Fig. 2:**
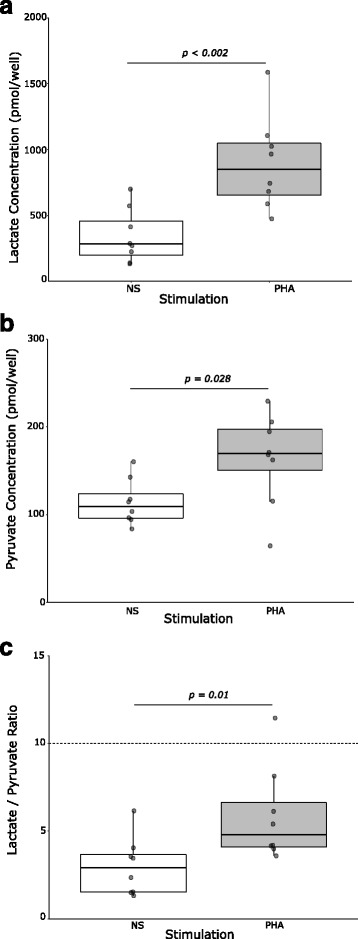
Effect of PHA-stimulation on intra-T cell metabolites concentration. T lymphocytes were purified from eight healthy donors’ blood. Cells were cultured in complete RPMI medium at a concentration of one million cells/mL and were stimulated with 4 μg/mL PHA (grey box) or left unstimulated (NS, white box). After a 40-h incubation, one million T cells were aliquoted and intra-lymphocyte lactate and pyruvate concentrations measured. Results are expressed as the concentration of lactate (Fig. 2a) or pyruvate (Fig. 2b) per well of the lecture plate, which contains 50 μL of the obtained cell lysate, or as the lactate/pyruvate ratio (Fig. 2c), which is known to reflect anaerobic metabolism when exceeding 10 (horizontal dotted line). Each point represents the mean of the duplicates for each donor. Data are also presented as Tukey boxplots. Bottom and top of the box represent the first and third quartiles, respectively. The horizontal bar in the box represents the median value. Lower and higher extremities of the whiskers respectively represent the lowest datum still within 1.5 inter-quartile range (IQR) of the lower quartile, and the highest datum still within 1.5 IQR of the upper quartile. Difference between NS and PHA groups was assessed with Wilcoxon test

We then sought to determine if we were able to identify Warburg effect—aerobic glycolysis—induction in T cells through increased lactate production. We thus measured intra-lymphocyte lactate and pyruvate concentrations after the previously determined 40-h incubation period, in 1 × 10^6^ T cells from four additional healthy volunteers. We observed a significant increase in both glycolysis-related metabolites in stimulated versus control samples (Fig. 2a–b). In addition, the lactate/pyruvate ratio, despite increasing after PHA stimulation, remained below 10 in both conditions (Fig. 2c).

3. Correlations between intra- and extra-lymphocyte lactate concentrations

After intracellular production, lactate is supposed to be released in culture supernatant. To evaluate such mechanism in our experimental conditions, we thus measured the correlation between intracellular and supernatant lactate concentrations after T cell stimulation.

**Fig. 3:**
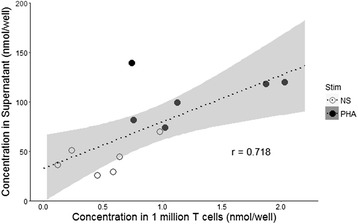
Correlation between intracellular and supernatant lactate concentration in healthy volunteer’s T lymphocytes. T lymphocytes were purified from six healthy donors’ blood. Cells were cultured in complete RPMI medium at a concentration of one million cells/mL, and stimulated with 4 μg/mL PHA (black circles) or left unstimulated (NS, open circles). After the previously determined 40-h incubation period, supernatant and one million T cells were aliquoted and lactate concentration was measured in supernatants and in T cells. Results are expressed as the quantity of lactate per well of the lecture plate, which contains 50 μL of the obtained cell lysate or supernatant. Each point represents the mean of the duplicates for each donor. Dotted line represents the linear regression model and grey zone represents the 95% confidence interval of the regression. Correlation between intra-lymphocyte and supernatant lactate concentration was explored with Pearson correlation

We cultured T cells from six healthy volunteers at 1 × 10^6^ cells/mL for 40 h, then harvested supernatants and cell pellets. Lactate concentrations were then measured in cell culture supernatant and in 1 × 10^6^ cultured T cells. We observed a good correlation (*r* = 0.72, 95% CI 0.24–0.91, *p* = 0.009) between intra and extracellular lactate concentrations (Fig. 3). Lactate concentration in culture supernatant was systematically 10-fold higher than lactate concentration measured intra-lymphocytes.

4. Minimal cell number

Profound lymphopenia is very common among critically ill patients, especially in those suffering from sepsis [18, 19]. Lowering the number of cells needed to show a difference in lactate concentration between control and stimulated T cells is therefore a critical point before a possible use in clinical settings.

**Fig. 4:**
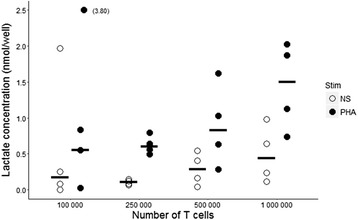
Minimal cell number for lactate measurement in healthy volunteers’ T lymphocytes. T lymphocytes were purified from four healthy donors’ blood. Cells were cultured in complete RPMI medium at a concentration of one million cells/mL and were stimulated with 4 μg/mL PHA (black circles) or left unstimulated (NS, open circles). After the previously determined 40-h incubation period, different number of T cells (ranging from 100,000 to 1,000,000) were aliquoted and intra-lymphocyte lactate concentrations measured. Results are expressed as the quantity of lactate per well of the lecture plate, which contains 50 μL of the obtained cell lysate. Each point represents the mean of the duplicates for each donor and horizontal bars represent the median value

After 40-h stimulation, lactate concentrations were measured in 100,000, 250,000, 500,000, and 1,000,000 T cells from 4 healthy donors. Lactate concentration was measurable even from 100,000 cells, either stimulated or not (Fig. 4). However, 250,000 T cells led to lower variability and can be obtained from a limited volume of septic patient’s blood. Higher lymphocyte numbers are very difficult to obtain within clinical constraints in septic shock patients, and did not show a clear additional benefit in terms of lower variability, compared to 250,000 cells. We thus chose to measure lactate concentration in 250,000 T lymphocytes from septic shock patients in further experiments, as it represented a compromise between, on the one hand, a measurable signal and difference between unstimulated and stimulated conditions, and on the other hand, clinical constraints.

II. Lactate production by lymphocytes from septic shock patients’ peripheral blood

After setting up the method for intra T lymphocytes lactate measurement, we conducted a pilot study on septic shock patients’ blood, in order to confirm the feasibility of this technique and to obtain preliminary exploratory data in patients.

1. Population description

We collected blood from seven patients at day 3 of septic shock, since previous data from our lab showed that sepsis-induced immunosuppression is maximal at this time-point [20, 21]. Control samples came from 10 healthy volunteers. Demographic and infection characteristics, as well as lymphocyte subsets distribution and other biological parameters are listed in Table 1. Day 28 mortality was 43% among septic shock patients. Patients displayed immunosuppressive phenotype, as demonstrated by a decreased mHLA-DR expression and profound CD4+ lymphopenia (Table 1).

**Table 1:** Septic shock patients’ characteristics (*n* = 7)

Age	73 [65–77]	Documentation of infection	
Male sex	4 (57%)	Clinics only	0 (0%)
Day 28 mortality	3 (43%)	Clinics + imaging	1 (14%)
		Clinics + surgery	1 (14%)
SAPS II score	62 [56–65]		
SOFA score	9 [8–12]	Microbiologically proved	5 (71%)
Charlson score	1 [0–3.5]	Gram-negative	4 (80%)
		Gram-positive	1 (20%)
Norepinephrine dosage			
At day 1 (μg/kg/min)	0.83 [0.54–1.78]	Lactatemia	
		Day 1	2.8 [2.3–3.8]
Mechanical ventilation	7 (100%)	Day 3	1.6 [1.4–1.6]
Type of admission		Lymphocyte purity	76.5 [69.2–85.9]
Medical	2 (29%)	CD3 purity	97.3 [96.6–97.9]
Surgical	5 (71%)		
		CD4 (%)	72.0 [59.9–80.9]
Type of infection		CD8 (%)	14.8 [11.0–26.5]
Community-acquired	2 29%)	CD4−/8− (%)	3.1 [1.8–8.2]
Nosocomial	5 (71%)		
		CD4 (/μL)	295 [220–413]
Site of infection		Treg (%)	7.7 [7.3–9.5]
Pulmonary	2 (29%)		
Intra-abdominal	3 (43%)	mHLA-DR (ab/c)	3079 [2590–4010]
Urinary	1 (14%)		
Others	1 (14%)		

*SAPS II* Simplified Acute Physiological Score II, *SOFA* sepsis-related organ failure assessment, Treg CD4 + CD25highCD127low regulatory T lymphocytes score, *mHLA-DR* monocyte surface expression of HLA-DR, *ab/c* number of antibodies bound per cell. Normal value for mHLA-DR is > 15,000 ab/c, indicative of immunocompetence (Docke, 2005). In-lab normal values for CD4+ T cells absolute count are 336–1126 cells/μL and 4–10% of total CD4+ lymphocytes for Treg. Values are expressed as number (percentage) for categorical variables and median [Q1–Q3] for continuous variables

2. Lactate concentration in peripheral T lymphocytes

**Fig. 5:**
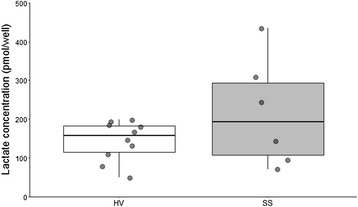
Intra-cellular lactate concentration in T lymphocytes in healthy volunteers versus septic shock patients. T lymphocytes were purified from 10 healthy donors’ and 7 septic shock patients’ blood. After the purification process, 250,000 T cells were aliquoted, lysed, and deproteinised. One septic patient was too lymphopenic to harvest 250,000 T cells, leading to one missing data. Results are expressed as the quantity of lactate per well of the lecture plate, which contains 50 μL of the obtained cell lysate. Each point represents the mean of the duplicates for each donor. Data are also presented as Tukey boxplots (white for HV, grey for SS). Bottom and top of the box represent the first and third quartiles, respectively. The horizontal bar in the box represents the median value. Lower and higher extremities of the whiskers respectively represent the lowest datum still within 1.5 inter-quartile range (IQR) of the lower quartile, and the highest datum still within 1.5 IQR of the upper quartile

Immediately after T lymphocyte purification process, intracellular lactate concentration was measured in 250,000 T cells from septic shock patients and healthy donors. We observed that intra-T lymphocyte lactate concentration was not different in T cells from septic shock patients compared with healthy donors (Fig. 5). Unfortunately, due to the low number of T cells that could be purified from septic patients’ blood, we were unable to measure intra-lymphocyte pyruvate concentration in parallel.

Interestingly, we observed that intra-lymphocyte lactate concentration was higher in day 28 non-survivors patients than in healthy volunteers, whereas it did not differ between survivors and controls (data not shown). This should not be regarded as a definitive conclusion, given the very low number of patients studied, but it would be of great interest to address this hypothesis in a future larger cohort.

## Discussion

Our work provides an optimized protocol for measuring lactate in human T lymphocytes. It deals with limitations encountered in clinical settings, such as lymphopenia, extensive apoptosis, and limited blood volume withdrawn [22]. We demonstrated that a very good correlation exists between lactate concentration in lymphocytes and in culture supernatants. To our knowledge, such correlation has not been described in human T cells in the literature so far. At last, we showed that such protocol can be used in sepsis-induced lymphopenic patients.

Stimulation of T lymphocytes from healthy volunteers induced lactate and pyruvate productions, suggesting the activation of glycolysis pathway. Cells were cultured in a non-hypoxic environment, with all nutrients in excess in the culture medium. That was confirmed by the low lactate/pyruvate ratio observed, below the threshold of 10 which is proposed as the normal value [23] and can be used in clinical settings to detect anaerobic metabolism. The observed activation of glycolysis cannot therefore be attributed to anaerobiosis. Thus, we reproduced experimentally the so-called Warburg effect, or aerobic glycolysis, ex vivo in isolated human T lymphocytes stimulated with PHA.

After being produced from glucose in the glycolysis pathway, lactate can either enter Krebs cycle, thus being converted into pyruvate, or diffuse through cell membrane via MCT-1 to 4 (monocarboxylate transporter) pores [24]. This transmembrane transport is driven by a concentration gradient. This gives physiological support to the correlation observed between intra-lymphocyte and supernatant concentrations of lactate, in healthy volunteers. Indeed, lactate concentration in culture supernatant was systematically 10-fold higher than lactate concentration measured into lymphocytes, suggesting that the major part of lactate produced by these cells is rapidly secreted in the extracellular milieu.

Glycolysis is triggered when the organism is subjected to any intense stimulus. Activation of glycolysis results in intense pyruvate synthesis. When the capacities of PDH to convert pyruvate into acetyl-CoA to enter the TCA cycle become exceeded, or if a mitochondrial dysfunction exists, pyruvate is then converted into lactate via LDH by the law of mass action. For example, a β2 stimulation with epinephrine induced important muscular release of lactate and pyruvate in different animal model of shock states, which was not related to oxygen availability [25].

Lymphocytes express β2-adrenergic receptors. Their precise functions remain unclear to date [26], but their ligation modulates cell behavior in a subset-specific manner [27] and seems to participate in the shift of Th pattern observed in T lymphocytes during septic shock [8]. However, stimulation of these receptors during sepsis, as well as lymphocyte activation triggered by the infectious agent, represents a rational for lactate production by lymphocytes during septic shock. Preferential use of glycolysis over oxidative phosphorylation may be a part of the metabolic reprogramming of immune cells that exists in sepsis [28].

Interestingly, lactate itself has immunomodulatory effects. In mice, lactate injection suppressed lymphocyte cytotoxic responses [29]. High lactate concentration in tumor microenvironment might affect immune cells functionality. In an ex vivo human study, lactate suppressed proliferation and cytokine production of cytotoxic T cells [30]. Such lymphocyte alteration was reproduced by MCT-1 pore blockade, suggesting that lactate export outside the cell is mandatory for such effector functions. As lactate flux through MCT-1 depends on a concentration gradient, increased lactate concentration in the tumor microenvironment might impair lactate output from lymphocytes, thus disturbing their metabolism and function [30]. Regarding innate immunity, lactate also inhibited TNF production by monocytes [31]. To note, immunosuppressive effect could be induced by both lactate presence and acidification of the milieu. In the study by Dietl et al. authors demonstrate that lactic acid but also, to a lesser extent, sodium lactate (a sodium salt of lactic acid, without effect on the pH) inhibit the secretion of TNF alpha by monocytes. At low lactic acid concentrations, both lactate and mild acidification are necessary; at higher concentrations, acidification seems to be the dominant factor [31]. In cancer, such effects of lactate could favor the local immune tolerance of the tumor by suppressing immune cells functionality. One can assume that lactate effects on immune cells competence might be identical during sepsis. To some extent, lactate production by lymphocytes followed by export in the milieu might worsen sepsis-induced lymphocyte alterations in a negative feedback loop.

Our study does not permit to conclude if, during septic shock, lymphocytes are responsible for lactate production, or if the increased plasma lactate might diffuse into lymphocytes through its membrane. However, lactate may have consequences on lymphocytes functionality during sepsis.

Serum lactate level has been repeatedly shown as a marker of the intensity of aggression and disease severity, and is proposed to reflect the intensity of tissue damage and peripheral organs dysfunctions. Lactate level and kinetics is a good biomarker for mortality prediction in septic shock but also in unselected critically ill patients [32–35]. Meanwhile, lactate production by T lymphocytes is one marker of these T cells’ bioenergetic status, interconnecting metabolism, and immune functionality (immunometabolism), as it has been shown recently in monocytes [7]. Thus, these two parameters are probably not evaluating similar dysfunctions after septic shock, although they might be linked to some extent.

Our study relies only on seven septic shock patients’ blood samples, representing its primary limit. Our first goal was to set up a protocol for lactate measurement in T lymphocytes, within the clinical constraints encountered with septic shock patients. However, we demonstrated its feasibility and obtained interesting preliminary results. It will be of great interest to confirm these exploratory findings in a larger cohort of septic shock patients.

## Conclusion

Intra-lymphocyte lactate concentration may represent the immune cell ability to respond to an intense stress, as such response is very energy-consuming and would therefore imply glycolysis. It would be of great interest to compare this technique to gold standard tests, for example, lymphocyte proliferation assay either by tritiated thymidine or EdU (5-ethynyl-2′deoxyuridine, a DNA base analog) incorporation [14] or cytokine production. Given the relationship between lactate and cell cycle, it would be very interesting to include cell cycle measurements in further prospective cohorts. The pathophysiology of the complex interactions between metabolism and immune functionality of lymphocytes should be further investigated as it may represent an interesting research field for innovative therapies.

## Abbreviations

CD: Cluster of differentiation
EDTA: Ethylenediaminetetraacetic acid
EdU: 5-ethynyl-2′deoxyuridine
FCS: Fetal calf serum
HV: Healthy volunteer
LDH: Lactate dehydrogenase
mHLA-DR: Monocytic human leukocyte antigen-DR
OVN: Overnight
PDH: Pyruvate dehydrogenase
PHA: Phytohaemagllutinin
RPMI: Roswell Park Memorial Institute
SS: Septic shock
TCA: Tricarboxylic acid (cycle)
Tregs: Regulatory T lymphocytes
